# Satisfying Outcomes Scores and Survivorship Achieved With Impaction Grafting for Revision THA in Young Patients

**DOI:** 10.1007/s11999-015-4293-y

**Published:** 2015-04-18

**Authors:** Martijn A. J. te Stroet, Wim H. C. Rijnen, Jean W. M. Gardeniers, Albert van Kampen, B. Willem Schreurs

**Affiliations:** Radboud University Medical Center, Geert Grooteplein 10, 6525 GA Nijmegen, The Netherlands

## Abstract

**Background:**

The increasing number of total hip arthroplasties (THAs) performed in younger patients will inevitably generate larger numbers of revision procedures for this specific group of patients. Unfortunately, no satisfying revision method with acceptable survivorship 10 years after revision has been described for these patients so far.

**Questions/purposes:**

The purposes of this study were to (1) analyze the clinical outcome; (2) complication rate; (3) survivorship; and (4) radiographic outcome of cemented revision THA performed with impaction bone grafting (IBG) on both the acetabular and femoral sides in one surgery in patients younger than 55 years old.

**Methods:**

During the period 1991 to 2007, 86 complete THA revisions were performed at our institution in patients younger than 55 years. In 34 of these 86 revisions (40%), IBG was used on both the acetabular and femoral sides in 33 patients. Mean patient age at revision surgery was 46.4 years (SD 7.6). No patient was lost to followup, but three patients died during followup. None of the deaths were related to the revision surgery. The mean followup for the surviving hips was 11.7 years (SD 4.6). We also analyzed complication rate.

**Results:**

The mean Harris hip score improved from 55 (SD 18) preoperatively to 80 points (SD 16) at latest followup (p = 0.009). Six hips underwent a rerevision (18%): in four patients, both components were rerevised; and in two hips, only the cup was revised. Patient 10-year survival rate with the endpoint of rerevision for any component for any reason was 87% (95% confidence interval [CI], 67%–95%) and with the endpoint of rerevision for aseptic loosening, the survival rate was 97% (95% CI, 80%–100%). In total six cups were considered radiographically loose, of which four were rerevised. Three stems were radiographically loose, of which none was rerevised.

**Conclusions:**

IBG is a valuable biological revision technique that may restore bone stock in younger patients. Bone stock reconstruction is important, because these patients likely will outlive their revision implants. Bone reconstruction with impaction grafting may facilitate future revisions.

**Level of Evidence:**

Level IV, therapeutic study.

## Introduction

The increasing number of THAs performed in patients younger than 55 years will inevitably generate a larger number of revision procedures for this specific group of patients. Unfortunately, data on the recent results of revision THA in younger patients are limited, and survivorship data from studies of younger patients are not encouraging [1, 16, 22]. The single long-term survival study reports a disappointing outcome of 63% at 10 years for patients when the endpoint was rerevision for any reason [22].

Although the use of highly crosslinked polyethylene and alternate bearings appears to have reduced the occurrence of severe osteolysis and bone loss, managing bone loss represents a substantial challenge in revision hip surgery. This is particularly true for patients with older designs of hip implants and for younger patients, because these patients have a long life expectancy and may require repeated revisions.

Impaction bone grafting (IBG) is a biological reconstruction technique that can restore bone defects [19, 33]. It was first described for the acetabulum in 1984 by Slooff et al. [31] and subsequently, for the femur, in 1993, by Gie et al. [14]. Satisfying results have been reported from several centers with up to 25 years of followup on the acetabular side [5, 9, 15, 29] and up to 20 years on the femoral side [13, 21, 26, 32, 34]. In our opinion, the IBG technique may be a valuable treatment option in revision THA in patients younger than 55 years.

We therefore chose to analyze the clinical outcome, complication rate, survivorship, and radiographic outcome of cemented revisions using impaction allografting on both acetabular and femoral sides in patients under the age of 55 years.

## Patients and Methods

The study was approved by our institutional review board. All patients were followed up prospectively, and the data were retrospectively analyzed. Only patients with a minimum followup of 5 years from one single academic center in The Netherlands were included.

Patients undergoing revision of failed primary THA inserted for oncologic reasons were excluded. It is our policy to perform IBG in combination with cemented components in all revisions with acetabular or femoral bone stock loss, including in younger patients.

During the period 1991 to 2007, 86 complete THA revisions were performed at our institution in patients younger than 55 years. In 34 of these 86 revisions (40%), IBG was used on both the acetabular and femoral sides in 33 patients. The 52 other revisions were performed without IBG or with only acetabular or femoral IBG.

The mean age of the 19 women and 14 men at the time of the revision surgery was 46.4 years (SD 7.6). One woman was operated on both hips. The mean weight of the patients at the time of the surgery was 78.4 kg (SD 16.7); mean height was 172 cm (SD 10); and the mean body mass index was 26.4 kg/m^2^ (SD 5.1). Eighteen procedures (55%) were performed on the right side.

At the time of our latest review (January 2013), no patient had been excluded or was lost to followup. Three patients died during the study—two after 4 years and one after 7 years of followup (9%); none of the deaths was related to the surgery and data for the three patients were included in our final analyses. The mean followup of the surviving hips was 11.7 years (SD 4.7).

The indication for the primary THA was trauma in 13 hips, osteoarthritis (OA) after childhood hip diseases in seven hips, primary OA in four hips, OA secondary to osteonecrosis in three hips, rheumatoid arthritis in three hips, septic coxitis in one hip, coxitis tuberculosa in one hip, and unknown for two hips.

The indication for revision was aseptic loosening of both components in 19 hips, septic loosening in 14, and, in one, stem malpositioning with aseptic loosening of the cup.

In 19 hips this was the first revision procedure, nine hips already were revised once or more in our department, and six hips were already revised in another hospital. All of these earlier revisions were for aseptic reasons.

It was the first acetabular revision in 22 hips, the second in 10, and the third in two. On the femoral side, it was the first revision in 26 hips and the second in eight hips.

Twelve of 14 hips with septic loosening were treated with a two-stage procedure; all patients were given systemic antibiotics guided by the intraoperative cultures for at least 6 weeks before reimplantation. The diagnosis of septic loosening in the remaining two hips was based on bacterial cultures taken during a one-stage revision for what had been thought to be aseptic loosening. These two patients also were treated with systemic antibiotics.

Four surgeons participated in this study; two of the authors (JWMG, BWS) performed 20 and 11 reconstructions, respectively.

The surgical technique of IBG has been described in detail before for both the acetabular [30] and femoral [28] sides. In all hips, a posterolateral approach was used. If infection was suggested, a two-stage revision was performed. Segmental acetabular and femoral bone defects were reconstructed using metal meshes and wires. In acetabular reconstructions, a medial wall mesh was used in three; in 15, a rim mesh; and in 10, a combination of both. Femoral reinforcement with a metal mesh, screws, and cerclage wires was used for the proximal femur in seven patients, the distal femur in one, and a combination of both in two patients. On the acetabular side, between one and four fresh-frozen femoral head allografts were used; and on the femoral side, between one and three. All femoral revisions were performed with the X-change^®^ femoral revision system (Stryker-Howmedica, Newbury, UK). In 30 reconstructions, a normal length, polished, tapered Exeter™ component (Stryker-Howmedica) was used, and in four, a longer Exeter component (≥ 205 mm) was required. The acetabular components used were a Muller^®^ cup (Sulzer, Wintherthur, Switzerland) in 12 hips, an Exeter™ RSA cup (Stryker-Howmedica) in eight hips, an Exeter Contemporary™ flanged cup in seven hips, an Exeter Contemporary™ hooded cup in six hips, and a Charnley^®^ Elite plus cup (DePuy, Leeds, UK) in one hip. Average duration of the reconstruction surgeries was 4.01 hours (range, 2.32–6.15 hours [ie, incision to close time]). The antibiotic-loaded bone cement used in all hips was Surgical Simplex^®^ (Stryker-Howmedica) containing erythromycin and colistin.

The postoperative drug regimen included systemic administration of antibiotics given after obtaining the cultures (three intravenous doses of 1 g cefazolin) for 1 day. Indomethacin was used for 7 days to prevent heterotopic ossification. All patients received anticoagulation therapy for at least 6 weeks.

The postoperative physiotherapy protocol changed during the years. In general, in the first revisions, patients were on bed rest for 6 weeks with passive movement of the operated hip starting 24 hours postoperatively. During the next 6 weeks, toe-touch weightbearing was allowed, and during the last 6 weeks, patients were permitted to load 50% body weight on the affected hip with use of two crutches. As a result of increased experience with the IBG technique, the duration of bed rest was reduced, and currently, patients start toe-touch weightbearing 1 day after surgery.

All patients were seen at 6 weeks, 3 months, 6 months, and 12 months postoperatively and then annually or biannually at our institution. All clinical and functional scores were obtained by an independent research assistant.

Clinical evaluation was prospectively performed using the Harris hip score (HHS: worst score 0, best score 100) [18], the Oxford hip score (OHS: worst score 0, best score 48) [25], and the visual analog scales (VAS) [3] for pain at rest and pain during physical activity—using a scale from 0 (no pain) to 100 (unbearable pain). All patient scores were determined during postoperative followup, and the HHS was also determined preoperatively. The scores provided here were from the latest followup visit.

A complete set of radiographs was available for 33 (97%) of the 34 hips. Bone stock defects were classified based on both preoperative radiographs and intraoperative findings using the classification as described by Paprosky et al. for the acetabular side [27] and by Della Valle and Paprosky for the femoral side [8] (Table 1). All radiographs were scored on a consensus basis by two of the authors (MAJS, BWS). Radiolucencies of ≥ 2 mm were scored around the acetabular component in the three zones of DeLee and Charnley [7] and on the femoral side with use of the seven zones described by Gruen et al. [17]. Migration or tilting of the acetabular component was estimated in relation to the line between the teardrops [12]. Femoral subsidence was determined with the method of Fowler et al. [10]. When radiolucent lines ≥ 2 mm wide were present in all three acetabular or seven femoral zones, component migration was ≥ 5 mm, and/or tilting was ≥ 5°, the component was considered radiographically loose. Trabecular incorporation was evaluated with use of the criteria of Conn et al. [6], defined as equal radiodensity of graft and host bone with a trabecular pattern throughout.

**Table 1:** Classifications—preoperative bone stock defects

Type	Definition	Number of hips at risk (%)
Acetabular defects as described by Paprosky et al. [27]		
1	Minimal destruction of the acetabular rim and bone lysis localized to cement anchor holes	3 (9)
2A	Generalized enlargement of the acetabulum with minimal osteolysis of the dome and slight superior and medial migration of the cup	7 (20)
2B	Similar to type 2A defects, but more destruction of the dome is present	14 (41)
2C	Defects involve destruction of the medial wall with generalized rim enlargement	3 (9)
3A	Bone loss patterns involve the superior rim of the acetabulum from the 10 o’clock to the 2 o’clock position and often display medial wall deficiency	4 (12)
3B	Similar to type 3A defects, but the rim defects span from 9 o’clock to the 5 o’clock position	3 (9)
Femoral defects as described by Della Valle and Paprosky [8]		
1	Minimal loss of metaphyseal cancellous bone with an intact diaphysis	8 (23)
2	Extensive loss of metaphyseal bone with an intact diaphysis	17 (50)
3A	The metaphysis is damaged severely and nonsupportive; a minimum of 4 cm of intact cortical bone is present in the femoral isthmus	6 (18)
3B	The metaphysis is severely damaged with some intact cortical bone present distal to the isthmus (< 4 cm)	1 (3)
4	Extensive metadiaphyseal damage in conjunction with a widened femoral canal	2 (6)

We performed Kaplan-Meier survivorship analyses, including 95% confidence intervals (CIs), for the complete revision THAs (acetabular and femoral component) considering any rerevision of one of the components a “failure.” The endpoints used were rerevision of one or both components for any reason, for aseptic loosening, and reoperation for any reason. Patient survival outcomes for all endpoints were also determined separately for both the acetabular and femoral components. We used Wilcoxon rank sum test to compare pre- and postoperative HHS.

## Results

### Clinical Outcomes Scores

The mean HHS had improved from 55 (SD 18) preoperatively to 80 points (SD 16) at latest followup (p = 0.009). The mean postoperative OHS was 36 (SD 10). The mean postoperative VAS score for pain at rest was 12 (SD 21) and for pain during exercise 26 (SD 31).

### Complications/Reoperations

#### Intraoperative Complications

Six of 34 (18%) revision procedures were complicated by an intraoperative fracture with two (6%) on the acetabular side during the impaction process and four (12%) on the femoral side during removal of the removal of the old stem or cement.

### Rerevisions, Reoperations, and Other Postoperative Complications

Nine of 34 hips (26%) underwent one or more reoperations after the index revision procedure, including six (18%) revision procedures and three (9%) irrigation and débridements. Of the nine procedures, four were performed for septic complications (12% of all hips), three were performed for aseptic component loosening (9%), one for traumatic loosening (3%), and one for recurrent instability (3%). Of the five nonseptic procedures, two were complicated by subsequent periprosthetic infection. During followup, a rerevision was performed in six patients (18%); in four patients, both components were rerevised, and in two patients, only the cup was revised (Table 2).

**Table 2:** Indications for rerevision in six patients

Components rerevised	Indication for rerevision	Followup (years)
4 rerevisions of both the acetabular and femoral component	1 aseptic loosening	11.7
	1 septic loosening (conversion to permanent excision arthroplasty)	3.6
	1 stem rerevision for malpositioning causing recurrent dislocations	1.6
	(with later rerevision of both components for septic loosening)	2.9
	1 cup rerevision for traumatic loosening after fall	8.7
	(with later rerevision of both components for septic loosening)	9.8
2 cup rerevisions	2 aseptic loosenings	2.6 and 10.8

Three patients underwent débridement and antibiotic therapy for reinfections after index two-stage revision performed for septic loosening. These three reconstructions are still in situ and the patients are functioning well, although they use suppressive antibiotics.

Two patients experienced dislocations. One patient was treated conservatively with success and the other patient was treated with a femoral revision for stem malpositioning with recurrent instability.

One patient had a fracture of the greater trochanter after a fall, which could be treated conservatively.

### Survivorship

**Fig. 1A–C:**
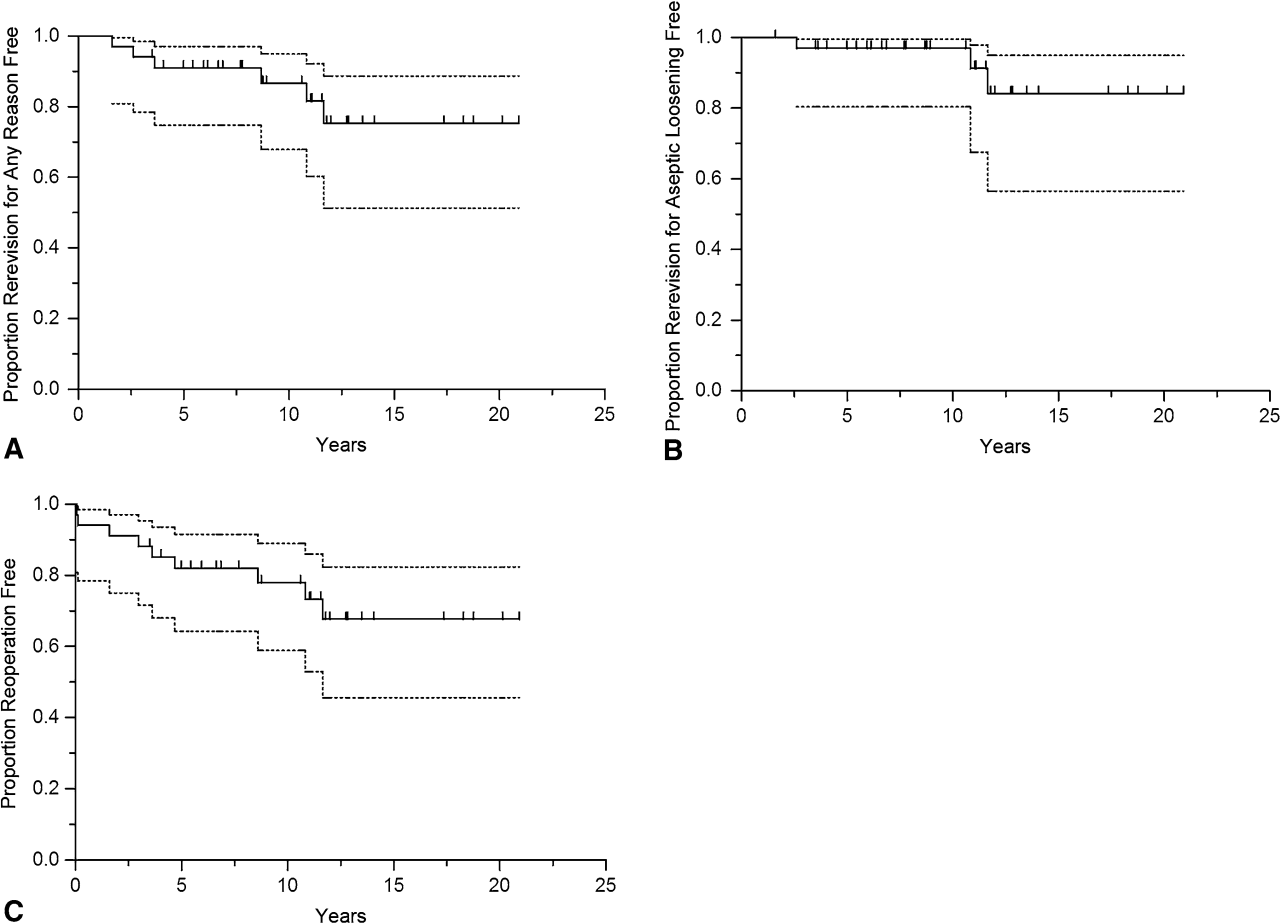
Kaplan-Meier survival curve shown for complete revision THAs (acetabular and femoral components) with (**A**) rerevision for any reason; (**B**) rerevision for aseptic loosening; and (**C**) reoperation for any reason of one or both components as the endpoint.

Kaplan-Meier analysis showed that the survival rate of the revision THA with rerevision of one or both components for any reason as the endpoint was 87% (95% CI, 67%–95%) at 10 years (Fig. 1A). With rerevision of one or both components for aseptic loosening as the endpoint, patient 10-year survival was 97% (95% CI, 80%–100%; Fig. 1B) and with reoperation for any reason as the endpoint 78% (95% CI, 59%–89%; Fig. 1C). We also analyzed the survival rate for different periods of followup and for the separate acetabular and femoral components (Table 3).

**Table 3:** Survival for different followup times and endpoints

Rerevision	5 years		10 years		15 years	
	Survival (95% CI)	Number of patients at risk	Survival (95% CI)	Number of patients at risk	Survival (95% CI)	Number of patients at risk
Survival complete revision THA (acetabular and femoral component)						
Rerevision for any reason (events = 6)	91% (75–97)	28	87% (68–95)	18	75% (51–89)	5
Rerevision for aseptic loosening (events = 3)	97% (80–100)	28	97% (80–100)	18	84% (57–95)	5
Reoperation for any reason (events = 9)	82% (64–92)	25	78% (59–89)	18	68% (46–82)	5
Survival acetabular components only						
Rerevision for any reason (events = 6)	91% (75–97)	28	87% (68–95)	18	75% (51–89)	5
Rerevision for aseptic loosening (events = 3)	97% (81–100)	28	97% (81–100)	18	84% (57–95)	5
Reoperation for any reason (events = 9)	82% (64–92)	25	78% (59–89)	18	68% (46–82)	5
Survival femoral components only						
Rerevision for any reason (events = 4)	94% (78–98)	29	89% (69–97)	18	83% (58–94)	6
Rerevision for aseptic loosening (events = 1)	100%	29	100%	18	93% (59–99)	6
Reoperation for any reason (events = 7)	85% (68–94)	26	81% (62–91)	18	75% (53–88)	6

CI = confidence interval.

### Radiographic Analysis

#### Acetabular Side

Radiolucencies were observed around 12 cups (35%). In four cups, radiolucencies were seen in all three zones; three of the four cups were rerevised; the fourth patient was chronically infected. Four cups had radiolucencies in two zones; one of these was rerevised. Five nonrerevised cups had radiolucencies in one zone.

In six hips, migration and/or tilting of the cup was observed; four of these were rerevised. The other two cups gradually tilted 5° and 13°, the latter, again, a chronically infected patient.

So, in total six cups were considered radiographically loose, of whom four were rerevised. In the nonrerevised acetabula, trabecular incorporation could be scored in 83 of 84 (99%) zones; one was obscured by a metal mesh. In 70 of 83 zones (84%), incorporation was seen.

#### Femoral Side

**Fig. 2A–C:**
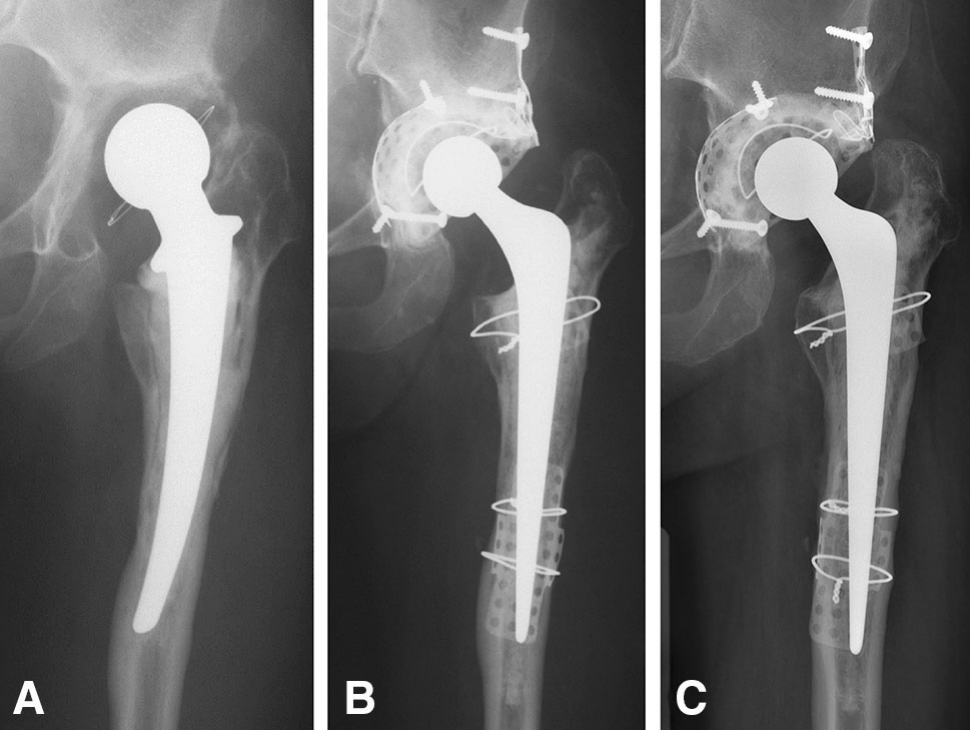
(**A**) Preoperative radiograph of a female patient, about to undergo THA, showing aseptic loosening and bone loss of both the acetabulum and proximal femur. The patient had previously undergone total cemented revision implantation. The revision components were in situ for 11 years. The patient was 19 years old when the primary total hip prosthesis was implanted after a coxitis. The primary THA was revised after 15 years. (**B**) Radiograph after the index revision surgery with IBG of both the acetabulum and the femur. The patient was 45 years old at the time of the revision. Segmental bone defects of the medial and lateral acetabular wall were reconstructed with metal meshes and screws. On the femoral side, a distal cortical perforation was covered with a metal mesh and cerclage wires, and the dorsal calcar region was also reconstructed. (**C**) Radiograph at final followup 19 years after the index revision with IBG. Both revision implants are stable with incorporation of the bone grafts, although a small osteolytic area can be seen in Gruen Zone 1 of the femur. The patient was 64 years old at last followup and still has an excellent functioning hip (HHS 95) 45 years after the first THA.

Radiolucencies were observed around 11 stems (32%); these were seen in one zone in five patients, in two zones in five patients, and in five zones in one patient. The mean subsidence of the stem within the cement mantle was 2.6 mm (range, 0–8.8 mm). Three stems subsided between 5 and 10 mm. In two of these, the subsidence was progressive. None of these three stems needed rerevision. In the nonrerevised hips, 198 of the total 210 Gruen zones (94%) could be scored for trabecular incorporation; 12 zones were obscured by metal meshes. Incorporation (Fig. 2) was seen in 181 of the 198 zones (91%).

## Discussion

The increasing number of THAs performed in younger patients will inevitably generate a larger number of revision procedures in this group of patients. This is the first study that describes satisfying results of revision THA performed in patients aged < 55 years. This study fills a gap in the revision THA literature, because the reported results in younger patients have been disappointing until now [1, 16, 22]. The 10-year survivorship was 97% for rerevision for aseptic loosening and 87% for rerevision for any reason.

This study has a number of limitations. First, it was impossible to radiographically evaluate trabecular incorporation in a number of acetabular (1%) and femoral (6%) zones that were treated with extensive reconstruction with meshes. However, we did not find a difference in clinical or survivorship outcomes for these cases. Additionally, also the evaluation if a component is radiographically loose, sometimes is difficult when using this revision technique. Nevertheless, with the criteria we defined for component loosening, we were able to assess the loosening of the components adequately. Second, we were missing patient preoperative OHS and VAS scores because during the early phase of the study, our structured preoperative screening was still in the developing stage. Third, our number of patients seems to be relatively small. Nevertheless, the clinical outcome of all patients had been reported, resulting in a loss-to-followup quotient of zero, which is, according to Murray et al. [24], ideal. Also the radiographic followup was nearly complete (97%). We chose to study only those patients who had complete IBG revision of both the acetabular and femoral sides in one surgery because the outcome of only a cup or stem revision will always be influenced by the component left in situ at the partial revision. Fourth, in the majority of the included revisions, no extensive bone stock defects were present. When we had included only patients with Paprosky grades > 3 on both the acetabular and femoral sides, the results would probably have been worse than now.

The mean HHS at final followup in our study had improved 25 points. This comparable to the improvement in clinical scores reported by other revision series performed in younger patients. Adelani et al. [1] reported a mean improvement in HHS of 19.2 points and Lee et al. [22] reported an improvement of 36 points in the modified HHS (no physical examination is necessary for this modified scoring system).

Regarding the postoperative complications, we reported a postoperative periprosthetic fracture in one patient (3%), dislocations in two patients (6%), and a reoperation for a deep infection in four patients (12%). Of the studies on the revision THAs in young patients, only Lee et al. [22] specified their postoperative complications. They observed 10 periprosthetic fractures (5.5%), 11 dislocations (6.1%), and two deep infections (1.1%). An explanation for the high percentage of reoperations for infections in our study could be the relatively high percentage of index revisions that were performed for septic loosening (14 procedures [41%]). Our hospital is a tertiary referral center for the treatment of prosthetic infections and complex hip surgery. As a result, in our study group of younger patients, the indication for primary THA in only four patients was standard with primary OA. Most patients had undergone more than one previous operation on their hip for traumatic, congenital, or other complex hip problems before the index revision was performed. The mean number of previous operations was 4.3 for the 14 septic revisions compared with two for the 20 aseptic revisions. This higher number of previous operations certainly can explain the high rate of infectious cases.

In two patients, an acetabular fracture occurred during the impaction process (6%). Once recognized, this problem can be solved by fracture fixation using an additional plate or mesh. These fractures have not deteriorated the outcome as was shown in our study. One of the patients functioned well but died 4 years after the revision; the other patient still has a stable reconstruction 9 years after surgery. Unfortunately, none of the previous revision studies in young patients specified their intraoperative complications making a comparison impossible.

When we compare the survival outcomes, the multicenter trial published by Girard et al. [16] reported a disappointing 10-year survivorship of 36% for the endpoint rerevision for any reason (77 revisions in 55 patients aged < 30 years). In only 21 revisions both components were exchanged and these patients were extremely young. Adelani et al. [1] performed a case-controlled study of 103 mostly uncemented revisions compared with 103 primary THAs. Patients accounting for 43 other revisions performed during the study period were lost to followup (29.5%). At a mean followup time of 6.7 years, 71 revisions (69%) survived compared with 102 (99%) primary THAs. However, only 11 of 103 revisions including both components were revised. Lastly, Lee et al. [22] recently published their results of 181, mostly uncemented revisions (of which 109 were complete revisions) in 102 patients aged 50 years or younger after a mean followup of 11 years. Unfortunately, 27 patients were lost to followup before the minimum of 2 years (and therefore only included in the survival analysis) and the radiographs of 67 patients were lost. The 10-year survival for endpoint rerevision for any reason was 63%. The authors suggested that bone grafting could be a reasonable option to further improve the outcome in this group of younger patients. It seems that the 10-year results of our cemented IBG revisions are at least comparable to the results of all these studies.

Radiological interpretation of graft incorporation in IBG remains difficult, despite the criteria defined by Conn et al. [6]. Incorporation can only be proven histologically. In a study based on 24 human acetabular biopsies taken between 1 month and 15 years after reconstruction, revascularization and incorporation of the bone graft were generally seen [33]. Most cases showed lamellar bone with few graft remnants. Also in a human retrieval, Heekin et al. [19] reported complete incorporation of morsellized allograft on the acetabular side. We could score radiographic signs of trabecular incorporation in 84% of the acetabular zones and 91% of the femoral zones in the surviving hips. In other studies performed with bone impaction grafting, Emms et al. [9] reported union of the graft in 64 of the 67 surviving acetabular reconstructions performed with irradiated allografts (95.5%) and Wraighte and Howard [34] reported radiographic evidence of trabecular incorporation in 87% of the Gruen zones that they could evaluate in 75 femoral bone impaction grafting revisions. Comparison of our radiographic results with the results of other revision series in young patients unfortunately is not possible, because in all these three studies, no radiographic results are described.

Next to IBG, other treatment modalities could be of use in revision THAs in young patients. In case of large acetabular defects jumbo cups [20] or modified cup shapes [4, 23] are possible treatment options. Also custom-made implants can be promising, although only short-term results have been reported until now [11]. In cases with femoral bone stock loss for example, cementless modular stems [2] can be inserted. However, when using all these techniques, the bone stock loss is not replenished and none of these techniques up until now have been reported to produce good results in a specific young patient group.

In conclusion, IBG is a valuable biological revision technique that may restore bone stock with satisfying long-term results and seems to be an especially beneficial option in younger patients because of their longer life expectancy. Younger patients will even outlive their revision implants and, hence, face future revisions. The amount of bone stock is essential to facilitate future revisions in these still relatively young patients.
